# Predicting head and neck cancer treatment outcomes with pre-treatment quantitative ultrasound texture features and optimising machine learning classifiers with texture-of-texture features

**DOI:** 10.3389/fonc.2023.1258970

**Published:** 2023-10-02

**Authors:** Aryan Safakish, Lakshmanan Sannachi, Daniel DiCenzo, Christopher Kolios, Ana Pejović-Milić, Gregory J. Czarnota

**Affiliations:** ^1^ Czarnota Lab, Physical Sciences, Sunnybrook Research Institute, Sunnybrook Health Sciences Centre, Toronto, ON, Canada; ^2^ Department of Radiation Oncology, Sunnybrook Health Sciences Centre, Toronto ON, Canada; ^3^ Department of Physics, Toronto Metropolitan University, Toronto, ON, Canada; ^4^ Department of Medical Biophysics, University of Toronto, Toronto, ON, Canada

**Keywords:** quantitative ultrasound, radiomics, texture analysis, texture-of-texture, head and neck cancer, response prediction, deep texture analysis

## Abstract

**Aim:**

Cancer treatments with radiation present a challenging physical toll for patients, which can be justified by the potential reduction in cancerous tissue with treatment. However, there remain patients for whom treatments do not yield desired outcomes. Radiomics involves using biomedical images to determine imaging features which, when used in tandem with retrospective treatment outcomes, can train machine learning (ML) classifiers to create predictive models. In this study we investigated whether pre-treatment imaging features from index lymph node (LN) quantitative ultrasound (QUS) scans parametric maps of head & neck (H&N) cancer patients can provide predictive information about treatment outcomes.

**Methods:**

72 H&N cancer patients with bulky metastatic LN involvement were recruited for study. Involved bulky neck nodes were scanned with ultrasound prior to the start of treatment for each patient. QUS parametric maps and related radiomics texture-based features were determined and used to train two ML classifiers (support vector machines (SVM) and *k*-nearest neighbour (*k-NN*)) for predictive modeling using retrospectively labelled binary treatment outcomes, as determined clinically 3-months after completion of treatment. Additionally, novel higher-order texture-of-texture (TOT) features were incorporated and evaluated in regards to improved predictive model performance.

**Results:**

It was found that a 7-feature multivariable model of QUS texture features using a support vector machine (SVM) classifier demonstrated 81% sensitivity, 76% specificity, 79% accuracy, 86% precision and an area under the curve (AUC) of 0.82 in separating responding from non-responding patients. All performance metrics improved after implementation of TOT features to 85% sensitivity, 80% specificity, 83% accuracy, 89% precision and AUC of 0.85. Similar trends were found with *k*-NN classifier.

**Conclusion:**

Binary H&N cancer treatment outcomes can be predicted with QUS texture features acquired from index LNs. Prediction efficacy improved by implementing TOT features following methodology outlined in this work.

## 1 Introduction

Cancers of the oral cavity, pharynx, larynx, paranasal sinuses, nasal cavity and salivary glands are broadly categorised as head and neck (H&N) cancers (1). The World Health Organization (WHO) estimated diagnosis of 930,000 new H&N cancer cases in the year 2020 (2) making them the 6^th^-most common type of cancer (3). Approximately, 90% of H&N cancers are squamous cell carcinomas (SCC) (4). Risk factors include tobacco (5) and alcohol consumption (6), p53 (7) and p16 gene mutations (8, 9) and the presence of human papilloma virus (HPV) genomic DNA (10). Distant metastasis is rare at the time of diagnosis (10%), but the majority of patients present with disease in regional lymph nodes (LNs) in addition to a primary location (3).

Treatment plans for H&N cancer patients typically include a combination of surgery, radiotherapy (XRT), and systemic therapy, and are often individualised depending on disease stage, as well as patients’ health at the time of treatment. Although different fractionation schemes are practiced for up-front radiotherapy, the standard objectives for H&N treatments typically include 70 Gy in 33-35 fractions to a high dose target volume for gross disease, and 63 Gy and 56 Gy in 33-35 fractions to intermediate and low dose (risk) target volumes, respectively (11). Advances in personalised patient care, including more accurate treatment-planning software and innovations like intensity modulated radiation therapy (IMRT) will likely continue to improve outcomes for patients (12). 5-Year mortality rates are dependent on both stage and location of tumours, with survival rates near 90% for lip cancers but below 40% for cancer of the hypopharynx (13). Despite considerable developments, there are a subset of patients who do not exhibit the desired response to treatment.

Tumour composition and microenvironments are widely studied with focus on trying to understand the mechanisms from which tumour masses exhibit heterogeneity (14). There seems to be evidence supporting the notion that increased intratumoural heterogeneity reduces the likelihood of successful response to treatment (15). Tumour heterogeneity lays the foundation for the emergence of resistance and eventually, potential disease relapse, as the cancer tumour is made up of cells with varying characteristics and responses to targeted treatment (15). Quantitative ultrasound spectroscopic (QUS) parameters have been shown to detect disorganisation of tissues (16–18). Exploring cancer treatment response with QUS features can shed light on characterising malignancies based on acoustic properties.

Diseases are associated with physical alterations in tissues that can cause observable changes in acoustic scattering properties (17– 19). With that premise, in 1987 Lizzi et al. published seminal work on the concept of quantitative ultrasound spectroscopy (QUS); this is an analytical approach to determine tissue “acoustic signatures” from the frequency content of the backscattered signal of US radiofrequency (RF) data, that are related to the effective sizes, concentrations, and acoustic impedances of tissue elements (19). From QUS power spectra, various spectral parameters, like mid-band fit (MBF), spectral slope (SS), and 0-MHz spectral intercept (SI) can be determined. In addition, two backscatter coefficient parameters, average scatter diameter (ASD) and average acoustic concentration (AAC) can also be evaluated using a Gaussian scattering model (20) and a fluid-filled sphere model (also referred to as Anderson model) (21). QUS Parameters can be used to effectively characterise various biological conditions, including but not limited to apoptosis (17), evaluating hepatic steatosis for patients with non-alcoholic fatty liver disease (22), differentiating hepatocellular carcinoma from at-risk and normal liver parenchyma (23), and benign and malignant thyroid nodules (24).

Previous works by A. Dietz & S. Delorme found a relationship between sonographically high vascularisation of LNs and a less favourable prognosis by investigating colour Doppler images and the correlation between lower relative colour pixel density (hypovascularisation) and more favourable outcomes (25, 26). Lin et al. (2013) found that when comparing QUS spectral parameters of mouse tumours treated with Adriamycin to control tumours, SS and MBF significantly increased (by 48% and 13%, respectively) in comparison to the control group (*p <* 0.001 and *p =* 0.013, respectively) (27). There have also been several investigations into breast cancer classification (28), monitoring (29) and prediction of treatment outcomes (30), using QUS parameters and QUS parametric map features.

QUS Parameters can be computed for small overlapping windows to create QUS parametric maps from which texture features can be determined using radiomics. The field of radiomics was pioneered by Haralick et al. (1973) and has since expanded due to improved imaging and computational techniques and hardware (31). Radiomics involves determining feature data from biomedical images based on the assumption that texture information may be represented by the overall or ‘average’ spatial relationship of pixels within images (31). In order to describe ‘average’ spatial relationships, Haralick et al. introduced the concept of the gray level co-occurrence matrix (GLCM), a newly formed matrix based on relationships between neighbouring pixels in an image (31). **Supplementary Figure 1** demonstrates an example GLCM calculation from a sample 5x5 pixel image of five distinct pixel intensities. Textural feature (contrast, homogeneity, entropy, etc.) calculations are defined for the GLCM and other similar matrices (gray level run length matrix (GLRLM) (32), gray level size zone matrix (GLSZM) (33), and the gray level dependence matrix (GLDM) (34). Texture feature values can be calculated for the entirety of an image, or for a region of interest (ROI). Once texture-based features are matched with retrospective treatment outcome labels, machine learning (ML) classifiers can be trained to create predictive models.

Predictive models capable of reliable and effective prediction of treatment outcomes could lead to marked improvements related to personalised cancer care. Patients predicted to respond well to treatment would be given reassurance about treatment efficacy and ease-of-mind to undergo treatment. Such technology would also serve to benefit patients predicted to not achieve desired outcomes by permitting treatment interventions such as changes in radiation dose or fractionation (e.g. dose escalation). Previously, Tran et al. (2019) investigated LN phenotypic signals associated with H&N cancer treatment outcomes in creating predictive models (35). Building on the aforementioned work, improvements were made to address some key limitations; mainly (i) increasing sample size from *n* = 32 to *n* = 72, and (ii) using GLCM, GLRLM, GLSZM, and GLDM features as opposed to solely GLCM features. In addition to addressing some of the limitations of the work by Tran et al. (2019), we also incorporated and evaluated the effectiveness novel higher-order texture-of-texture (TOT) features in improving ML model performance for predicting treatment response. This study investigated the utility of quantitative ultrasound (QUS) texture-based features in predicting the treatment response of H&N cancer patients with metastatic LNs. Furthermore, the effect of potentially enhancing predictive models by implementing novel, higher-order texture-of-texture (TOT) features was evaluated.

## 2 Materials and methods

### 2.1 Study procedures and treatment

This study was approved by the institutional Research Ethics Board. Subjects (*n* = 72) were recruited and enrolled with informed written consent obtained prior to participation. Subjects had biopsy-confirmed diagnosis of H&N cancers being treated with radiotherapy for gross disease, and pathologically enlarged and measureable LNs (detailed below). Participants in this study had a median age of 61 years (ranging 36-82 years old). The mean age at the time of diagnosis was 60 years with a majority (*n =* 67, 93%) being males. Although there is a large discrepancy between male and female subjects, it should be noted that a 25-year analysis of cancer prevalence in Canada revealed that out of nearly 48,000 total H&N cancers, 70% (~35,000) were males (36). Smoking status, drinking status, primary tumour staging, histological analysis, and HPV status were also noted when available. 62 Patients (*n* = 62, 86%) were treated with chemotherapeutics (cisplatin, carboplatin, cetuximab, and carboplatin + etoposide) and the remaining ten (*n =* 10) were treated with definitive radiation alone. **Table 1** summarises patient, disease, and treatment characteristics for all subjects. **Supplementary Table 1** shows a breakdown of tumour and treatment characteristics for each patient involved.

**Table 1:** Patient characteristics for 72 patients in this study.

Patient Characteristics	*n* (%)
Patient and Clinical Characteristics *n* = 72 (all subjects)	
Age (years)	
- Median	- 61
- Mean	- 60.5 ± 10.14
Gender	
- Male	- 67 (93.1)
- Female	- 5 (6.9)
Smoking Status:	
- Smoker	- 48 (66.7)
- Non-smoker	- 23 (31.9)
- Unknown	- 1 (1.4)
Drinking Status:	
- Drinker	- 51 (70.8)
- Non-drinker	- 15 (20.8)
- Unknown	- 6 (8.3)
Tumour status	
Primary Tumour(T):	
- T1	- 4 (5.6)
- T2	- 23 (32)
- T3	- 7 (9.7)
- T4	- 15 (20.8)
- Unknown	- 23 (32)
Histological Type:	
- Squamous cell carcinoma	- 67 (93)
- Small cell carcinoma	- 1 (1.4)
- Nasopharyngeal carcinoma	- 4 (5.5)
HPV status:	
- p16(+)	- 41 (56.9)
- p16(-)	- 2 (2.8)
- Unknown	- 29 (40.3)
Treatment Characteristics	
Chemotherapy	- 62 (86.1)
- Cisplatin	- 55 (76.4)
o Low dose	- 2 (2.8)
o Medium dose	- 45 (62.5)
o High Dose	- 8 (11.1)
- Carboplatin	- 5 (6.9)
- Carboplatin + etoposide	- 1 (1.4)
- Cetuximab	- 1 (1.4)
No Chemotherapy	- 10 (13.9)
Post Treatment (3-month assessment from MRI)	
Complete Responder – locoregional control (CR)	- 25 (34.7)
Partial Responder – locoregional failure (PR)	- 47 (65.3)

Gross tumour volume (GTV) segmentations were expanded by 5 mm to form the high-dose clinical target volume on the primary and nodal volume. Furthermore, a 1 cm margin was added to the GTV to create the clinical tumour volume (CTV56) volume. XRT Administration was carried out using IMRT or volumetric modulated arc therapy (VMAT) techniques available at Odette Cancer Centre, Sunnybrook Health Sciences Centre, in Toronto, Ontario, Canada. In order to have been considered pathologically enlarged and measurable, a LN must have been ≥ 15 mm in “short axis” when assessed by computed tomography (CT) scan (with CT scan slice thickness recommended no greater than 5 mm). At baseline and in follow-up, only the short axis was evaluated and measured. Nodal size is normally reported as two dimensions in the plane in which the image is obtained (for CT scan this is almost always the axial plane; for magnetic resonance imaging (MRI) the plane of acquisition may be axial, sagittal, or coronal). The smaller of these measures is the “short axis”.

Patients were labeled as complete or partial responders (CR or PR) based on clinical follow-up using contrast enhanced MR imaging (based on *Response Evaluation Criteria in Solid Tumours* (RECIST) guidelines) conducted in the first three months after completion of treatment (37). Through visual inspection patients were categorised as CR if the index LN was found to be <1 cm, otherwise labeled as PR. Standard treatment protocol includes additional follow-ups every 3-6 months for the first two years, and every 6-12 months thereafter. Some patients may be “late responders” (PR group in the first three months, then CR at some later time point), however in this work the interest was in predicting response within the first three months.

### 2.2 Ultrasound data acquisition

The largest metastatic LN was identified on a diagnostic CT scan by a radiologist and referred to as the “index” LN. The index LN was scanned at various time points during treatment (baseline, 24 hours, 1 week, 4 weeks, and 7 weeks). In this study features were determined from the baseline scans which were acquired up to 2 weeks before starting treatment. The collected data included both grayscale (B-mode) images and the digitised (RF) signals. Data collection was from participating patients between 2015-2019, using an US device (Ultrasonix Med. Corp., BC, Canada). A linear 2D transducer (L4-5/38 Linear 4D, Ultrasonix) was used for imaging and RF-data collection, which had a centre frequency of approximately 10 MHz and a sampling rate of 40 MHz. Data was acquired across the entire LN volume, along 256 lateral scan lines (in-plane; 3.8 cm lateral field of view) with maximum axial depth of 5 cm. To account for the depth of the LN, the acoustic focus was adjusted for each patient individually (average depth = 1.75 cm).

### 2.3 QUS parameter determination

For each patient, segmentations were made, outlining the LN from six equally spaced B-mode images with associated RF-data using in-house MATLAB software. Following the procedures outlined by Lizzi et al. (1987), QUS spectra were computed using individual RF lines, by first applying a Hamming window before computing a fast Fourier transform (FFT) to determine the frequency component of the signal (19). An average power spectrum was then computed as the mean of the squared spectral magnitudes before calibrating it by dividing with a power spectrum of a tissue-mimicking phantom with known acoustic properties to remove various frequency dependent transfer functions and beam forming effects associated with the transducer (19).

**Figure 1:**
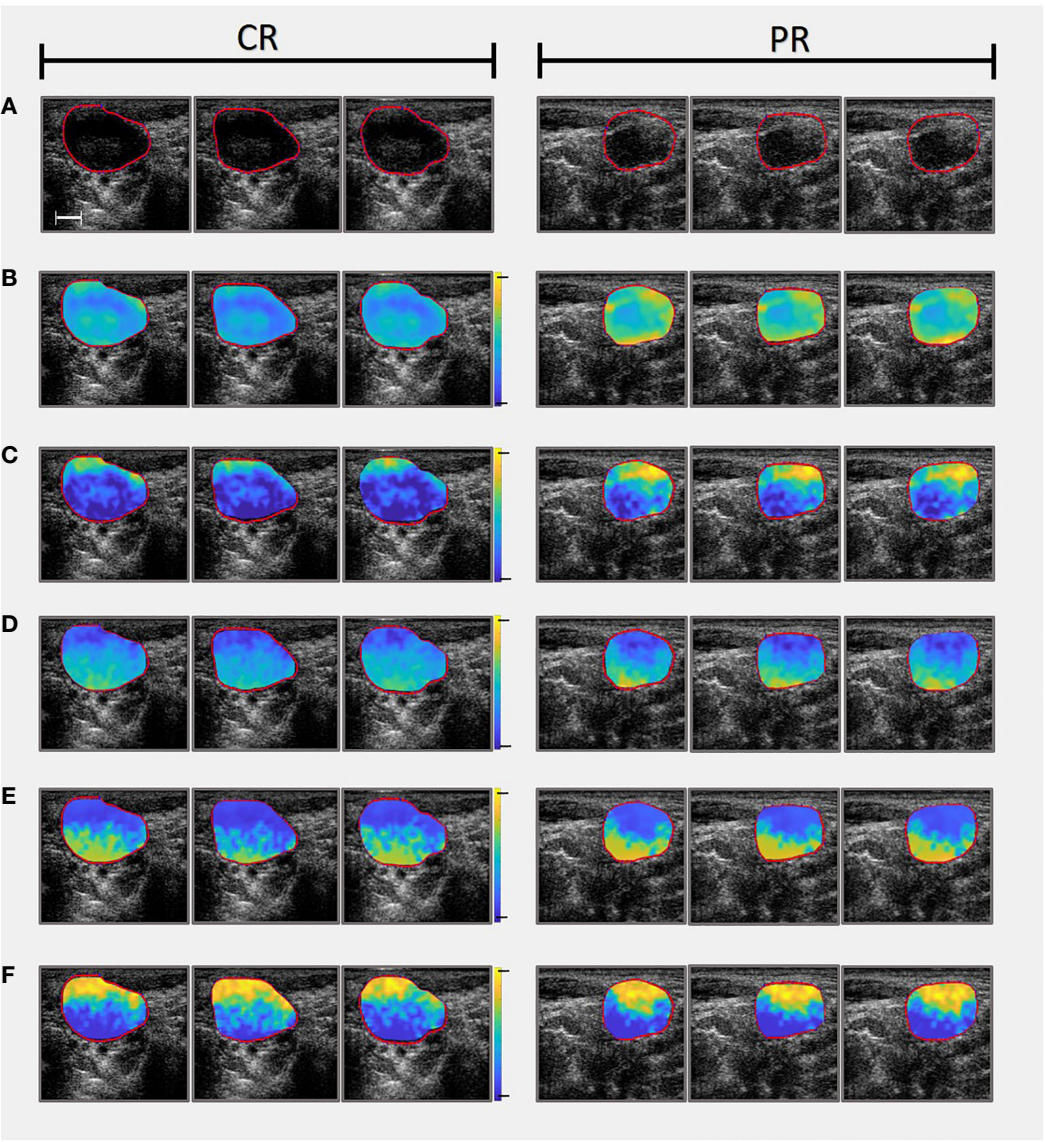
Comparing parametric maps (used as images to determine textural features from) and US slices of one CR patient (On the left) and one PR patient (On the right). **(A)** Presents the B-mode ultrasounds and accompanying LN ROIs. **(B)** Mid-Band Fit parametric maps (range from -10 to 40 dB). **(C)** Spectral Intercept parametric maps (range from 10 to 50 dB). **(D)** Spectral Slope parametric maps (range from -5 to 5 dB/MHz). **(E)** Average Acoustic Concentration calculated using the Gaussian model (range from 20 to 220 dB/cm^3^). Note that Average Acoustic Concentration was also calculated using Anderson model but omitted from this figure for convenience). **(F)** Average Scatterer Diameter calculated using the Gaussian model (range from 0 to 150 µm). Note that Average Scatterer Diameter was also calculated using Anderson model but omitted from this figure for convenience). Scale bar is 5 mm.

Linear regression analysis was performed on the normalised power spectrum to find the best-fit line within a -5 dB window (bandwidth of 3 – 8 MHz) centred at the transducer frequency . From the best-fit line, MBF, SS, and SI were computed. Additionally backscatter coefficient parameters ASD and AAC were determined from both a Gaussian model (20) and the fluid-filled model (also referred to as Anderson model) (21) for purposes of comparison. Local attenuation coefficient estimates (38) were used to calculate attenuation correction based on point-compensation method (39). QUS Parametric maps were created for seven QUS spectral parameters, using a sliding window technique with a window block of 2x2 mm and a 94.1% overlap between adjacent windows in both axial and lateral directions. In **Figure 1** representative QUS parametric maps (used to determine texture features) are shown for a CR and a PR patient, respectively.

### 2.4 Texture features

Texture features were determined from the QUS parametric maps using Pyradiomics, an open-source Python (Python Software Foundation, Delaware, USA) package (40). Features were determined from GLCMs (31) as well as other matrices (since developed to build on to the work of Haralick & colleagues), including GLRLM (32), GLSZM (33), and GLDM (34). For each of the seven QUS parametric maps, 68 features were determined (22 GLCM, 14 GLDM, 16 GLRLM, & 16 GLSZM features) for a total of 476 features per patient. The patient texture feature values were averaged across each tumour and matched with binary treatment outcomes, retrospectively. The dataset was used to train a predictive ML model to distinguish CR from PR patients. Details regarding ML modeling will be described in section 2.6.

### 2.5 Texture-of-texture

**Figure 2:**
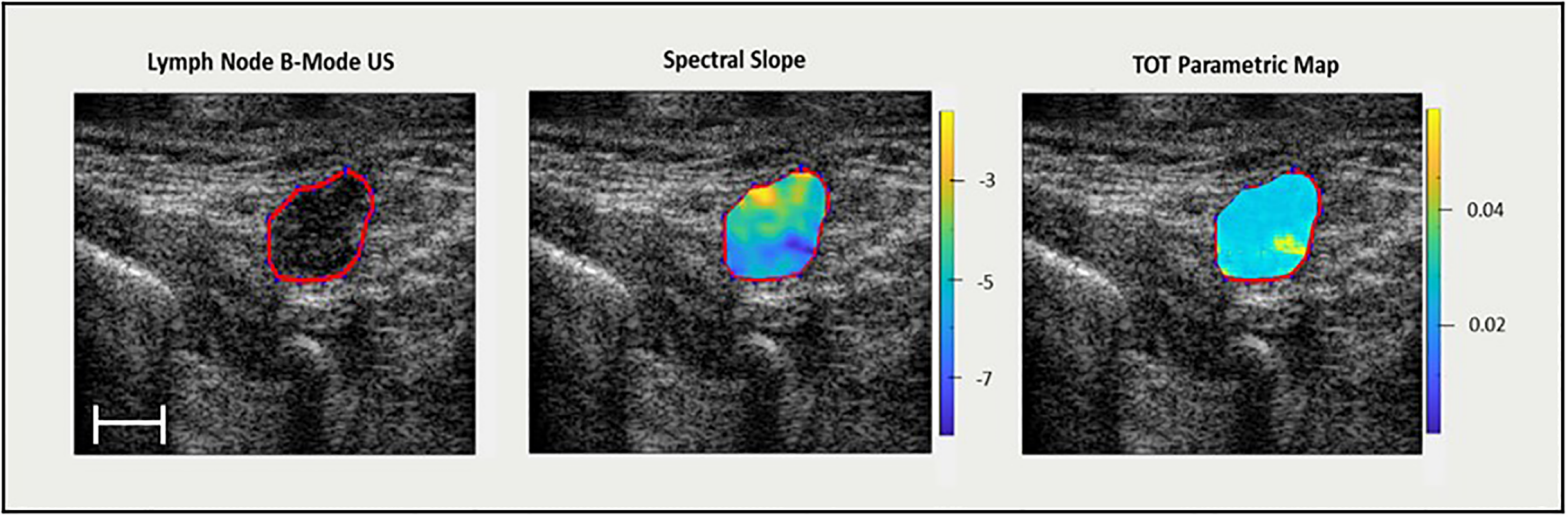
On the left, an US B-mode scan slice with lymph node ROI highlighted in red. In the middle, the QUS spectral slope parametric map (dB/MHz). On the right, a texture-feature parametric map (spectral slope – GLSZM – small area low gray level emphasis) (arbitrary units) from which TOT features are determined. Scale bar is 5 mm.

After preliminary model building, in order to enhance the performance of the classifiers, the effect of incorporating higher-order texture features was investigated. Higher-order texture features were calculated by creating texture-based images from which subsequent additional textures were determined. Informative features first determined in 5-feature multivariable models (process described below) were used as a guide to create new parametric maps, as presented in **Figure 2**. New texture parametric maps were created with a sliding window technique from smaller 3x3 pixel windows spanning the ROI.

As earlier, texture features were determined using Pyradiomics from the newly formed texture parametric maps and used along with the original five features to create a new dataset of features for classifier training. In addition to the GLCM, GLRLM, GLDM, and GLSZM features, first order statistics features related distribution of pixel intensities within the QUS texture parametric map. The same ML classifier parameters were used to train the classifiers with the new dataset to investigate whether performance was enhanced, and if texture-of-texture features contributed to the outcomes.

### 2.6 Machine learning processing

Mean QUS parameter values were calculated from ROIs for each image and subsequently averaged between all six tumour images to represent the entire LN mass. The differences of means between the two groups (CR/PR) were determined by computing the *p*-value in a one-tail test with results presented in **Table 2**.

**Table 2:** Comparing QUS parameters for entire ROI of CR vs PR group and comparing respective p-value for each of the seven parameters parametric maps were created for.

QUS Parameter (units)	ROI Mean Value (CR)	ROI Mean Value (PR)	*p*-Value (1-tail)
MBF (dB)	3.474	0.622	0.02
SS (dB/MHz)	-2.172	-2.050	0.30
SI (dB)	15.44	11.92	0.03
ASD_Gaussian (μm)	82.82	77.98	0.24
AAC_Gaussian (dB/cm^3^)	63.96	61.25	0.30
ASD_Anderson (μm)	142.9	136.7	0.10
AAC_Anderson (dB/cm^3^)	112.9	106.7	0.05

To account for data imbalances and bias from the majority class (35% CR/65% PR) and to avoid anomaly-type’ classification problems (41), a synthetic minority oversampling technique (SMOTE) was used in the pre-processing phase (41). To split the data, a leave-one-out cross validation method and 5 *k*-fold training set validation was implemented. Additional pre-processing included *z*-score scaling to account for varying magnitudes of texture feature values.

Feature selection was carried out by an iterative sequential forward selection (SFS) in a wrapper framework based on F1 score. Model performance was evaluated based on sensitivity (%S_n_), specificity (%S_p_), accuracy (%Acc), F1 score, precision, balanced accuracy, receiver-operating characteristic (ROC) curve, as well as area under the curve (AUC), for single-variable and multivariable models up to and including seven features as determined by either support vector machines (SVM) or *k-*nearest neighbour (*k*-NN) classifiers. Next, features identified in the five-feature multivariable model were used to create five new texture parametric maps using the sliding window technique. The reason for choosing five features to create parametric maps as opposed for example, to seven, was to keep computation time somewhat practical, as the creation of each texture parametric map can take hours (depending on ROI size) and must be computed for a total of 432 ultrasound slices (6 images per patient x 72 patients). Radiomic features of new texture parametric maps were determined using Pyradiomics to create a new set of texture-of-texture (TOT) features. New TOT features and initially selected five QUS texture features were used to create a second data set which was used to train ML classifiers to explore potential improvement of prediction efficacy. **Figure 2** presents a ROI labelled selected on a reconstructed US B-mode image, the corresponding QUS spectral slope parametric map, as well as a texture parametric map of a feature from a 5-feature multivariable model (spectral slope – GLSZM – Small Area Low Gray Level Emphasis). Creating QUS parametric maps, as well as ML classification, were carried out with MATLAB (Mathworks, MA, USA).

## 3 Results

### 3.1 Mean QUS values

**Table 2** presents QUS mean values for whole LN ROIs and compares CR and PR groups. A one-tailed t-test using a confidence level of *p* < 0.025 demonstrated the MBF parameter to be significantly different between the two groups (*p* = 0.020). The SI parameter showed a near-significant difference (*p* = 0.026) between the two groups, just missing the *p* < 0.025 confidence level. The remaining parameters, SS (*p* = 0.302), ASD_Gaussian (*p* = 0.241), AAC_Gaussian (*p* = 0.304), ASD_Anderson (*p* = 0.098), and AAC_Anderson (*p* = 0.049) were statistically insignificant between the CR and PR groups.

### 3.2 Treatment outcomes prediction from QUS texture features

Both ML algorithms used (SVM & *k*-NN) demonstrated an ability to predict treatment outcomes as summarised in **Table 3**. The SVM classifier model out-performed the *k*-NN classifier model with nearly every metric and combination of features (up to 7-feature multivariable model). The SVM classifier model performed best with a 6-feature model (%S_n_ = 80%, %S_p_ = 80%, %Acc = 81%, precision = 88% and AUC = 0.81). The *k*-NN classifier performed best with a 5-feature multivariable model (%S_n_ = 72%, %S_p_ = 72%, %Acc = 72%, precision = 83% and AUC = 0.72).

**Table 3:** Results from two SVM classifiers trained on QUS texture features for models with 1-7 features.

Features Used	%S_n_	%S_p_	%Accuracy	%Precision	AUC
SVM					
1	65.9	80.0	70.8	86.1	0.71
2	72.3	76.0	73.6	85.0	0.74
3	74.5	76.0	75.0	85.4	0.75
4	76.6	72.0	75.0	84.7	0.75
**5**	**78.7**	**76.0**	**77.8**	**86.1**	**0.82**
6	80.1	80.0	80.6	88.4	0.81
7	80.9	76.0	79.2	86.4	0.82
*k*-NN					
1	63.8	64.0	63.9	76.9	0.71
2	59.6	72.0	63.9	80.0	0.73
3	70.2	72.0	70.1	82.5	0.71
4	59.6	72.0	63.9	80.0	0.72
**5**	**72.3**	**72.0**	**72.2**	**82.9**	**0.72**
6	72.3	64.0	69.4	79.1	0.68
7	61.7	68.0	63.9	78.4	0.66

Features selected for models in bolded rows were used as guideline to create TOT features.

### 3.3 Model enhancement with TOT features

For both algorithms, selected features from the 5-feature multivariable model were used to create QUS-texture parametric maps. For the SVM classifier, these five features were “ASD_Anderson GLSZM Zone Entropy”, “SI GLDM Small Dependence Low Gray Level Emphasis”, “SS GLDM Small Dependence High Gray Level Emphasis”, “AAC_Gaussian GLDM Dependence Variance”, and “AAC_Anderson GLDM Small Dependence Emphasis”.

For the *k*-NN classifier, the five features used to create new parametric maps were “ASD_Anderson GLSZM Zone Entropy”, “ASD_Anderson GLRLM Long Run Low Gray Level Emphasis”, “ASD_Anderson GLSZM Large Area Low Gray Level Emphasis”, “ASD_Gaussian GLDM Low Gray Level Emphasis”, and “SS GLSZM Small Area Low Gray Level Emphasis”.

Texture features were determined for the newly created parametric maps and subsequently used along with the original 5 best features to create a new dataset with a total of 355 features (5 initially selected QUS texture features, and 350 higher-order TOT features) to be analyzed by ML classifiers. Identical classifier settings were used to evaluate the impact of incorporating higher order texture features in conjunction with top texture features. The results comparing QUS texture features to QUS texture + TOT features for SVM classifier are summarised in **Table 4**.

**Table 4:** Comparing SVM classifier results from texture-features from 5 (first row) and 7 (third row) features vs. 5 (second row) and 7 (fourth row) TOT features to evaluate improvement in prediction performance.

	SVM Classifier				
Features Used	%S_n_	%S_p_	%Accuracy	%Precision	AUC
5 Features QUS	78.7	76.0	77.8	86.1	0.82
5 Features QUS + TOT	78.7	80.0	79.2	88.1	0.85
7 Features QUS	80.9	76.0	79.2	86.4	0.82
**7 Features QUS + TOT**	**85.1**	**80.0**	**83.3**	**88.9**	**0.85**

In bold is the model that performed best with SVM classifier.

The implementation of TOT features improved the performance in classifying between CR and PR. Comparing the results of initial 5-feature multivariable model (all QUS texture features) to the 5-feature multivariable model (5 QUS texture features + TOT features) from the second data set with inclusion of TOT features for the SVM classifier demonstrated no change in sensitivity, however specificity, accuracy, precision and AUC improved (from 76% to 80%, 78% to 79%, 86% to 88%, and 0.82 to 0.85, respectively). Comparing the two 7-feature multivariable models, sensitivity improved from 81% to 85%, specificity increased from 76% to 80%, accuracy increased from 79% to 83%, precision increased from 86% to 89% and finally AUC increased from 0.82 to 0.85.

**Table 5** shows the results from the *k*-NN classifier. Comparing the sets of data with a 5-feature multivariable model, the introduction of TOT features increased sensitivity (from 72% to 78%), accuracy (from 72% to 74%) and AUC (from 0.72 to 0.75), however specificity, and precision decreased (from 72% to 64% and 83% to 80%, respectively). Similarly, for the 7-feature multivariable model, sensitivity, accuracy, precision, and AUC increased, and specificity remained unchanged.

**Table 5:** Comparing K-NN classifier results from texture-features from 5 (first row) and 7 (third row) features vs. 5 (second row) and 7 (fourth row) TOT features to evaluate improvement in prediction performance.

	k-NN Classifier				
Features Used	%S_n_	%S_p_	%Accuracy	%Precision	AUC
5 Features QUS	72.3	72.0	72.2	82.9	0.72
5 Features QUS + TOT	78.7	64.0	73.6	80.4	0.75
7 Features QUS	61.7	68.0	63.9	78.4	0.66
**7 Features QUS + TOT**	**74.4**	**68.0**	**72.2**	**81.4**	**0.75**

In bold is the model that performed best with k-NN classifier.

**Figure 3:**
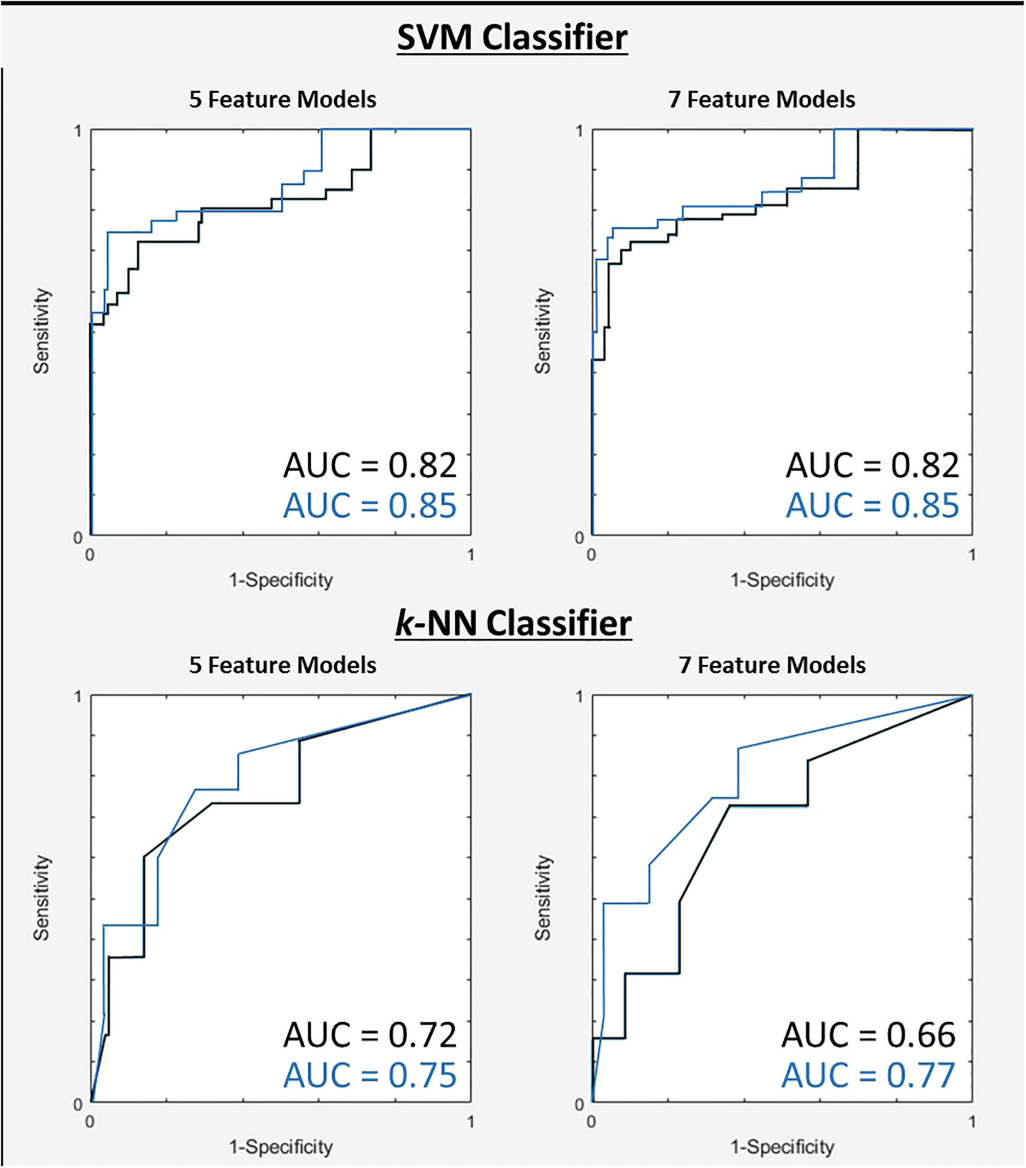
ROC curves for two tested classifiers for both 5 and 7-feature models trained on first dataset (black) and the second dataset after TOT features introduced (blue).

**Figure 4:**
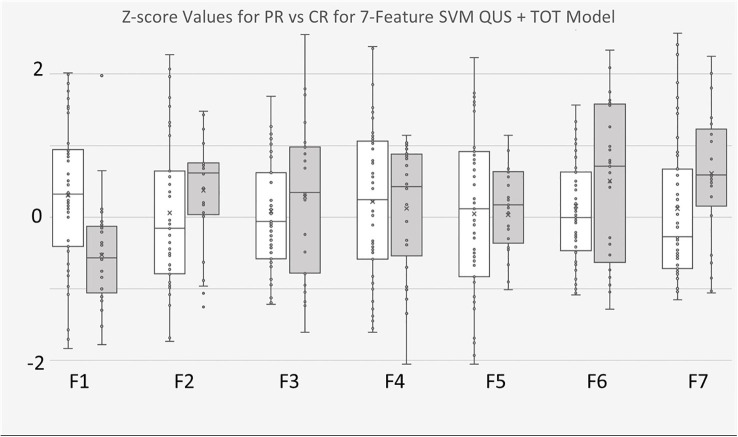
Boxplots of Z-score values for selected features of 7-feature SVM model trained on for QUS + TOT Features dataset. PR Patients shown in pink, CR patients shown in blue. F1 feature was “ASD_Anderson GLSZM Zone Entropy”. F2 feature was “SI GLDM Low Gray Level Emphasis”. F3 feature was AAC_Anderson GLDM Small Dependence Emphasis”. F4 feature was “SS GLDM Small Dependence High Gray Level Emphasis”. F5 feature was a TOT feature named “SS GLDM Small Dependence High Gray Level Emphasis GLSZM Size Zone Non-Uniformity Normalize”. F6 feature was also a TOT feature called “AAC_Anderson GLDM Small Dependence Emphasis GLCM Autocorrelation”. Finally, F7 feature was also TOT feature called “SI GLDM Small Dependence Low Gray Level Emphasis GLCM Cluster Prominence”.

The 7-feature multivariable model trained on QUS texture features + TOT features dataset yielded the best results, for both classifiers. **Figure 3** shows the ROC curves corresponding to the results from **Tables 4**, **5**. Interestingly, for the 7-feature multivariable SVM classifier model trained on the second dataset, of the seven selected features, four were among the five initial QUS texture features that were concatenated with the TOT features. These features were “ASD_Anderson GLSZM Zone Entropy”, “SI GLDM Small Dependence Low Gray Level Emphasis”, “AAC_Anderson GLDM Small Dependence Emphasis”, and “SS GLDM Small Dependence High Gray Level Emphasis”. The remaining three features were from the newly created TOT features, namely, “SS GLDM Small Dependence High Gray Level Emphasis GLSZM Size Zone Non-Uniformity Normalized”, “AAC_Anderson GLDM Small Dependence Emphasis GLCM Autocorrelation”, and “SI GLDM Small Dependence Low Gray Level Emphasis GLCM Cluster Prominence”. The distribution of these features between CR and PR patients can be seen in **Figure 4**.

**Figure 5:**
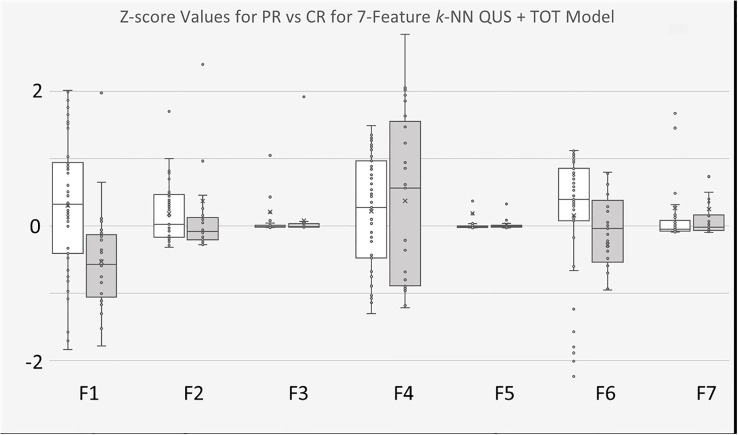
Boxplots of Z-score values for selected features of 7-feature k-NN model trained on for QUS + TOT Features dataset. PR Patients shown in white, CR patients shown in gray. F1 feature was “ASD_Anderson GLSZM Zone Entropy”. F2 feature was “ASD_Anderson GLSZM Large Area Low Gray Level Emphasis”. F3 feature was AAC_Anderson GLRLM Long Run Low Gray Level Emphasis”. F4 feature was “SS GLSZM Small Area Low Gray Level Emphasis”. F5 feature was a TOT feature named “ASD_Anderson GLRLM Long Run Low Gray Level Emphasis First Order 90 Percentile”. F6 feature was also a TOT feature called “ASD_Anderson GLSZM Zone Entropy GLSZM Zone Entropy”. Finally, F7 feature was also TOT feature called “ASD_Anderson GLRLM Long Run Low Gray Level Emphasis First Order Robust Mean Absolute Deviation”.

Similar outcomes were observed with the *k*-NN classifier models, wherein of the seven selected features from model trained with the second dataset, four were from the five preliminary QUS texture features, namely “ASD_Anderson GLSZM Zone Entropy”, “ASD_Anderson GLSZM Large Area Low Gray Level Emphasis”, “ASD_Anderson GLRLM Long Run Low Gray Level Emphasis”, and “SS GLSZM Small Area Low Gray Level Emphasis”. The remaining 3 selected features were newly created TOT features, namely “ASD_Anderson GLRLM Long Run Low Gray Level Emphasis First Order 90 Percentile”, “ASD_Anderson GLSZM Zone Entropy GLSZM Zone Entropy”, and “ASD_Anderson GLRLM Long Run Low Gray Level Emphasis First Order Robust Mean Absolute Deviation”. The distribution of these features between CR and PR patients can be found in **Figure 5**.

## 4 Discussion

In this study, it was found that pre-treatment QUS scans obtained from index LNs of H&N cancer patients may yield insight about clinical treatment endpoints. Additionally, a methodology was proposed to improve ML prediction model performance by implementing TOT features (results shown in **Tables 4**, **5**).

In the work here ASD and AAC QUS backscatter parameters were determined using both the Gaussian model (20) and fluid-filled, Anderson model (21). The rationale behind this decision was due to the nature of H&N LNs, which are both fluid-filled (favouring Anderson model) and displaying near spherical shape (favouring Gaussian model). Feature selection was used to remove redundant features and reduce dimensionality during classification. The best performance from both ML classifiers explored in this study (SVM and *k*-NN) was a 7-feature multivariable model based on a combination of four QUS-texture features and three higher-order TOT features. Despite the fact that all five originally identified QUS texture features were available for feature selection in the enhancement step, one was not selected, in favour of three TOT features. This suggests that the introduction of TOT features yielded additional phenotypic information related to treatment outcomes about LNs that are otherwise inaccessible solely through QUS-texture features. Interpretation of TOT features is challenging but should not be overlooked and are posited to be related to repetitive structures in bulky nodal structures and may be sensitive to a heterogeneity of structures.

Both classifiers identified “ASD_Anderson GLSZM Zone Entropy” as the most insightful feature. Zone Entropy is a Pyradiomics texture feature determined from the GLSZM matrix, and measures the uncertainty, or randomness, in the distribution of zone sizes and gray levels with higher values indicating more heterogeneous texture patterns (40). “Zones” in GLSZM refer to consecutive (or connected) pixels (in any direction including diagonal) which share identical pixel intensity values (33). Therefore, the GLSZM defines pixel relationships by considering zones of varying pixel intensities and sizes. Identification of “ASD_Anderson GLSZM Zone Entropy” as a significant feature suggests that pre-treatment heterogeneity in terms of diameter of acoustic scatterers within index LNs of H&N cancer patients may play an important role for response to treatment for H&N cancer patients.

For the model based on *k*-NN classifier, six of the seven selected features were based on ASD_Anderson parametric maps and one from SS parametric map. One feature, “ASD_Anderson GLSZM Large Area Low Gray Level Emphasis” measures the proportion of the joint distribution of large size zones with lower gray-level values in the image. Keeping in mind that this is a texture feature extracted from ASD parametric maps, it may be that larger areas of small scatterer diameters within the LN play a role in treatment efficacy. Another feature, “ASD_Anderson GLRLM Long Run Low Gray Level Emphasis” measures the joint distribution of long run lengths with low gray-level values. Once again, the feature was extracted from ASD_Anderson parametric maps, suggesting that longer run lengths [see GLRLM documentation for “run length” definition (32)] of low gray levels (smaller scatterer diameters) of index LNs plays a role in predicting treatment response. The final QUS texture feature selected in the 7-feature *k*-NN model is the GLSZM feature “Small Area Low Gray Level Emphasis” based on the SS parametric map, which is expected to be related to scatterer size (19). The remaining three features are TOT features, two based on ASD_Anderson GLRLM Long Run Low Gray Level Emphasis parametric maps, and one from ASD_Anderson GLSZM Zone Entropy parametric maps. The improved results demonstrate that TOT features provide finer information for the predictive model, which leads to better discrimination between CR and PR patients.

For the model based on SVM classifier, one selected feature was “GLDM Small Dependence Low Gray Level Emphasis” extracted from the SI parametric map. The GLDM considers the relationship between neighbouring pixels in all directions [see GLDM documentation for details (34)]. “Small Dependence Low Gray Level Emphasis” is a GLDM feature which measures the joint distribution of small dependence with lower gray-level values (40). Theoretically, SI is related to acoustic concentration and scatterer diameter and relative acoustic impedances of scattering elements (19). This suggests that within the SI parametric map, regions of sparsely distributed, lower spectral intercept values may play a role dictating treatment response. Another selected feature was the GLDM feature “Small Dependence Emphasis” extracted from AAC_Anderson QUS parametric maps. “Small Dependence Emphasis” is a measure of the distribution of small dependencies, with higher values indicative of smaller dependence and less homogeneous textures (40). This suggests heterogeneity of average acoustic concentrations within the LN may provide insight regarding treatment efficacy. The final QUS texture feature selected in the SVM classifier model was “GLDM Small Dependence High Gray Level Emphasis” extracted from SS parametric maps. “Small Dependence High Gray Level Emphasis” measures the distribution of small dependence with higher gray-level values (40). SS is a QUS parameter related to scatterer size (19), suggesting that small regions of higher slope values with in the LN plays a role in treatment efficacy. The final three features are TOT features extracted from “SS GLDM Small Dependence High Gray Level Emphasis”, “AAC_Anderson GLDM Small Dependence Emphasis”, and “SI GLDM Small Dependence Low Gray Level Emphasis” parametric maps. Once again, the improved results in discriminating between CR and PR patients suggests that TOT features provide finer information otherwise unavailable.

Other studies have looked into the effectiveness of radiomics features from various imaging modalities in predicting biological endpoints related to H&N cancers, for example, Tang et al. reported contrast-enhanced CT radiomics features acquired pre-treatment to be useful in predicting recurrence of locally advanced esophageal squamous cell carcinomas (42). Another investigation by Dang et al. reported MRI texture features to be promising in predicting p53 status in H&N squamous cell carcinomas (43). In the present study, work was built on previous findings from Tran et al. who reported results on prediction of H&N cancer treatment outcomes using just nine QUS GLCM features (35). The work here has improved on the work of Tran et al. by increasing the number of patients (from *n* = 32 to *n* = 72), the number of features computed for selection (from 41 to 476) and expanded beyond just GLCM features to GLCM + GLRLM+ GLSZM + GLDM features. Furthermore, in this study the effect of implementing TOT features was considered for improving response prediction by the ML algorithms. Finally, Tran et al. used logistic regression, Naïve Bayes, and *k*-NN classifiers, whereas in this study SVM and *k*-NN classifiers were investigated.

Due to the difference in sample size and the curse of dimensionality (44), Tran et al. considered a maximum of three features for their multivariable analysis, whereas in this study models with a maximum of seven features were evaluated. Of the three ML classifiers explored, Tran et al. report a best performance with sensitivity of 85%, specificity of 84%, accuracy of 88%, and AUC of 0.91 from the regression classifier with the 3-feature multivariable model (35). The best result from the study here was from the SVM classifier with sensitivity of 85%, specificity of 80%, accuracy of 83%, precision of 89%, and AUC of 0.85, which was the 7-feature QUS texture features + TOT features dataset multivariable model. The study here demonstrates a more robust and reliable model compared to previous work, mainly because of a larger sample size, and consideration of additional features.

Finally it is worth mentioning that patients enrolled in this study had to undergo ultrasound scans (not required for their treatment), solely for the purpose of advancing scientific research, which can present a challenge when recruiting vulnerable patients. Though results were promising, the relatively small sample size of this study suggests that these models are not generalizable for clinical applications. Furthermore, predictive models can incorporate clinical features, such as HPV status, to bolster features used to train models. However in this study only the feasibility of radiomics features were tested, as clinical features were not consistently available for all patients, because many patients received diagnostic work from outside institutions. Despite the limitations, the results are consistent with previous work, as well as promising, particularly when considering the possibility to improve results with the introduction of TOT features.

## 5 Conclusion

The study here was designed based on the hypothesis that the index LNs of H&N cancer patients contain acoustic phenotypes that can be correlated to the treatment response of the primary tumour and nodal disease. Insights regarding treatment responses using QUS texture features can potentially improve understanding of cancerous microstructures and provide another (non-invasive) tool at the disposal of clinicians in the aim of delivering the best personalised care to patients. Accurate and reliable predictions about treatment responses work to assist patients that fall into either CR or PR group. For example, patients who are predicted to respond well to treatment (CR) can be encouraged to forego any reservations they might be having of undergoing treatment. Fear of treatment can stem from risk of failure to cure, but also from the physical toll and decrease of quality of life. Patients predicted not to respond well to treatment (PR), can avoid undergoing ineffective treatment and the undesirable side effects associated with it and have altered therapy. Ultimately a better understanding of individual responses to a given treatment will benefit patients and continue to build on the path of personalised medicine.

## Data availability statement

The raw data supporting the conclusions of this article will be made available by the authors, without undue reservation.

## Ethics statement

The studies involving humans were approved by Sunnybrook Research Ethics Board. The studies were conducted in accordance with the local legislation and institutional requirements. The participants provided their written informed consent to participate in this study.

## Author contributions

AS: Data curation, Formal Analysis, Methodology, Visualisation, Writing – original draft, Writing – review & editing. LS: Software, Writing – review & editing. DD: Data curation, Writing – review & editing. CK: Software, Writing – review & editing. AP-M: Supervision, Writing – review & editing. GC: Conceptualisation, Funding acquisition, Investigation, Methodology, Supervision, Writing – review & editing.

## References

[1] Head and neck Cancers. National Cancer Institute (Bethesda, Maryland, USA: United States Department of Health and Human services). (2017). Available at: https://www.cancer.gov/types/head-and-neck/head-neck-fact-sheet.

[2] Global Cancer Observatory. International agency for Research on Cancer. Available at: https://gco.iarc.fr/today/online-analysis-table?v=2020&mode=cancer&mode_population=continents&population=900&populations=900&key=asr&sex=0&cancer=39&type=0&statistic=5&prevalence=0&population_group=0&ages_group%5B%5D=0&ages_group%5B%5D=17&group_cancer=1&i.

[3] Argiris A Karamouzis MV Raben D Ferris RL. Head and neck cancer. Lancet (2008) 371:1695–709. doi: 10.1007/174_2017_32 PMC7720415 18486742

[4] Skarsgard DP Groome PA Mackillop WJ Zhou S Rothwell D Dixon PF. Cancers of the upper aerodigestive tract in Ontario, Canada, and the United States. Cancer (2000) 88(7):1728–38. doi: 10.1002/(sici)1097-0142(20000401)88:7<1728::aid-cncr29>3.0.co;2-7 10738233

[5] Vineis P Alavanja M Buffler P Fontham E Franceschi S Gao YT. Tobacco and cancer: Recent epidemiological evidence. J Natl Cancer Inst (2004) 96(2):99–106. doi: 10.1093/jnci/djh014 14734699

[6] Blot WJ McLaughlin JK Winn DM Austin DF Greenberg RS Preston-Martin S. Smoking and drinking in relation to oral and pharyngeal cancer. Cancer Res (1988) 48(11):3282–7. 3365707

[7] Hejmadi M. Introduction to Cancer Biology. Ventus Publishing (2013) 2(4):2001. doi: 10.1517/14656566.2.4.613

[8] Dok R Glorieux M Holacka K Bamps M Nuyts S. Dual role for p16 in the metastasis process of HPV positive head and neck cancers. Mol Cancer (2017) 16(1):1–6. doi: 10.1186/s12943-017-0678-8 28662664 PMC5492443

[9] Bryant AK Sojourner EJ Vitzthum LK Zakeri K Shen H Nguyen C. Prognostic role of p16 in nonoropharyngeal head and neck cancer. J Natl Cancer Inst (2018) 110(12):1393–9. doi: 10.1093/jnci/djy072 PMC6292787 29878161

[10] McKaig RG Baric RS Olshan AF. Human papillomavirus and head and neck cancer: Epidemiology and molecular biology. Head Neck (1998) 20(3):250–65. doi: 10.1002/(SICI)1097-0347(199805)20:3<250::AID-HED11>3.0.CO;2-O 9570632

[11] Chau L McNiven A Arjune B Bracken G Drever L Fleck A. Dose objectives for head and neck IMRT treatment planning recommendation report. Cancer Care Ontario (2014), 1–10. Available at: https://www.cancercareontario.ca/sites/ccocancercare/files/guidelines/full/DoseObj_HN_IMRT_TrtmtPlngRec_0.pdf.

[12] Moreno AC Frank SJ Garden AS Rosenthal DI Fuller CD Gunn GB. Intensity modulated proton therapy (IMPT) – The future of IMRT for head and neck cancer. Oral Oncol (2019) 88:66–74. doi: 10.1016/j.oraloncology.2018.11.015 30616799 PMC6615027

[13] Cooper JS Porter K Mallin K Hoffman HT Weber RS Ang KK. National cancer database report on cancer of the head and neck: 10-year update. Wiley Period Inc (2009) 31(6):748–58. doi: 10.1002/HED 19189340

[14] Denison TA Bae YH. Tumor heterogeneity and its implication for drug delivery. J Control Release (2012) 164(2):187–91. doi: 10.1016/j.jconrel.2012.04.014 PMC3421061 22537887

[15] Dagogo-Jack I Shaw AT. Tumour heterogeneity and resistance to cancer therapies. Nat Rev Clin Oncol (2018) 15(2):81–94. doi: 10.1038/nrclinonc.2017.166 29115304

[16] Lizzi FL Astor M Feleppa EJ Shao M Kalisz A. Statistical framework for ultrasonic spectral parameter imaging. Ultrasound Med Biol (1997) 23(9):1371–82. doi: 10.1016/S0301-5629(97)00200-7 9428136

[17] Kolios MC Czarnota GJ Lee M Hunt JW Sherar MD. Ultrasonic spectral parameter characterization of apoptosis. Ultrasound Med Biol (2002) 28(5):589–97. doi: 10.1016/S0301-5629(02)00492-1 12079696

[18] Luchies AC Ghoshal G O’Brien WD Oelze ML. Quantitative ultrasonic characterization of diffuse scatterers in the presence of structures that produce coherent echoes. IEEE Trans Ultrason Ferroelectr Freq Control (2012) 59(5):893–904. doi: 10.1109/TUFFC.2012.2274 22622974 PMC3428796

[19] Lizzi FL Ostromogilsky M Feleppa EJ Rorke MC Yaremko MM. Relationship of ultrasonic spectral parameters to features of tissue microstructure. IEEE Trans Ultrason Ferroelectr Freq Control (1986) 34(3):319–29. doi: 10.1109/T-UFFC.1987.26950 18291854

[20] Insana MF Wagner RF Brown DG Hall TJ. Describing small-scale structure in random media using pulse-echo ultrasound. J Acoust Soc Am (1990) 87(1):179–92. doi: 10.1121/1.399283 PMC2745727 2299033

[21] Anderson VC. Sound scattering from a fluid sphere. J Acoust Soc Am (1950) vol:426–31. doi: 10.1121/1.1906621

[22] Pirmoazen AM Khurana A Loening AM Liang T Shamdasani V Xie H. Diagnostic performance of 9 quantitative ultrasound parameters for detection and classification of hepatic steatosis in nonalcoholic fatty liver disease. Invest Radiol (2022) 57(1):23–32. doi: 10.1097/RLI.0000000000000797 34049335

[23] Durot I Sigrist RMS Kothary N Rosenberg J Willmann JK El Kaffas A. Quantitative ultrasound spectroscopy for differentiation of hepatocellular carcinoma from at-risk and normal liver parenchyma. Clin Cancer Res (2019) 25(22):6683–91. doi: 10.1158/1078-0432.CCR-19-1030 31444249

[24] Goundan PN Mamou J Rohrbach D Smith J Patel H Feleppa EJ. A preliminary study of quantitative ultrasound for cancer-risk assessment of thyroid nodules. Front Endocrinol (Lausanne) (2021) 12:627698. doi: 10.3389/fendo.2021.627698 34093429 PMC8170470

[25] Dietz A Delorme S Rudat V Zuana I Conradt C Vanselow B. Prognostic assessment of sonography and tumor volumetry in advanced cancer of the head and neck by use of Doppler ultrasonography. Otolaryngol Neck Surg (2000) 122(4):596–601. doi: 10.1067/mhn.2000.98175 10740188

[26] Delorme S Dietz A Rudat V Zuna I Bahner ML Van Kaick G. Prognostic significance of color Doppler findings in head and neck tumors. Ultrasound Med Biol (1997) 23(9):1311–7. doi: 10.1016/S0301-5629(97)00153-1 9428129

[27] Lin C-Y Cao L-H Wang J-W Zheng W Chen Y Feng Z-Z. Ultrasonic spectrum analysis for *in vivo* characterization of tumor microstructural changes in the evaluation of tumor response to chemotherapy using diagnostic ultrasound. BMC Cancer (2013) 13(1):1. doi: 10.1186/1471-2407-13-302 23800247 PMC3698196

[28] Hsu SM Kuo WH Kuo FC Liao YY. Breast tumor classification using different features of quantitative ultrasound parametric images. Int J Comput Assist Radiol Surg (2019) 14(4):623–33. doi: 10.1007/s11548-018-01908-8 30617720

[29] Sannachi L Gangeh M Tadayyon H Gandhi S Wright FC Soldkowska E. Breast cancer treatment response monitoring using quantitative ultrasound and texture analysis: comparative analysis of analytical models. Transl Oncol (2019) 12(10):1271–81. doi: 10.1016/j.tranon.2019.06.004 PMC6639683 31325763

[30] Taleghamar H Jalalifar SA Czarnota GJ Sadeghi-Naini A. Deep learning of quantitative ultrasound multi-parametric images at pre-treatment to predict breast cancer response to chemotherapy. Sci Rep (2022) vol:1–13. doi: 10.1038/s41598-022-06100-2 35145158 PMC8831592

[31] Haralick RM Dinstein I Shanmugam K. Textural features for image classification. IEEE Trans Syst Man Cybern (1973) SMC-3(6):610–21. doi: 10.1109/TSMC.1973.4309314

[32] Chu A Sehgal CM Greenleaf JF. Use of gray value distribution of run lengths for texture analysis. Pattern Recognit Lett (1990) 11(6):415–9. doi: 10.1016/0167-8655(90)90112-F

[33] Thibault G Fertil B Navarro C Pereira S Cau P Levy N. Texture indexes and gray level size zone matrix application to cell nuclei classification. Int J Pattern Recognit Artif Intell (2013) 27(1). doi: 10.1142/S0218001413570024

[34] Sun C Wee WG. Neighboring gray level dependence matrix for texture classification. Comput Vision Graph Image Process (1983) 23(3):341–52. doi: 10.1016/0734-189X(83)90032-4

[35] Tran WT Suraweera H Quaioit K Cardenas D Leong KX Karam I. Predictive quantitative ultrasound radiomic markers associated with treatment response in head and neck cancer. Futur Sci OA (2019) 6(1). doi: 10.2144/fsoa-2019-0048 PMC6920736 31915534

[36] Canadian Cancer Statistics Advisory in collaboration with the Canadian Cancer Society, Statistics Canada and the Public Health Agency of Canada. Canadian Cancer Statistics: A 2022 special report on cancer prevalence. Toronto, ON: Canadian Cancer Society (2022). Available at: https://www.cancer.ca/Canadian-Cancer-Statistics-2022-EN.

[37] Eisenhauer EA Therasse P Bogaerts J Schwartz LH Sargent D Ford R. New response evaluation criteria in solid tumours: Revised RECIST guideline (version 1.1). Eur J Cancer (2009) 45(2):228–47. doi: 10.1016/j.ejca.2008.10.026 19097774

[38] Labyed Y Bigelow TA. Estimating the total ultrasound attenuation along the propagation path by using a reference phantom. J Acoust Soc Am (2010) 128(5):3232–8. doi: 10.1121/1.3483739 PMC3003735 21110618

[39] Oelze ML O’Brien WD. Frequency-dependent attenuation-compensation functions for ultrasonic signals backscattered from random media. J Acoust Soc Am (2002) 111:2308–19. doi: 10.1121/1.1452743 12051451

[40] Griethuysen JJMv Fedorov A Parmar C Hosny A Aucoin N Narayan V. Computational radiomics system to decode the radiographic phenotype. Cancer Res (2017) 77(21):e104–e107. doi: 10.1158/0008-5472.CAN-17-0339 29092951 PMC5672828

[41] Elreedy D Atiya AF. A Comprehensive Analysis of Synthetic Minority Oversampling Technique (SMOTE) for handling class imbalance. Inf Sci (Ny) (2019) 505:32–64. doi: 10.1016/j.ins.2019.07.070

[42] Tang S Ou J Wu YP Li R Chen TW Zhang XM. Contrast-enhanced CT radiomics features to predict recurrence of locally advanced oesophageal squamous cell cancer within 2 years after trimodal therapy A case-control study. Med (United States) (2021) 100(27):1–7. doi: 10.1097/MD.0000000000026557 PMC8270616 34232198

[43] Dang M Lysack JT Wu T Mathews TW Chandarana SP Brockton NT. MRI texture analysis predicts p53 status in head and neck squamous cell carcinoma. Am J Neuroradiol (2015) 36(1):166–70. doi: 10.3174/ajnr.A4110 PMC7965921 25258367

[44] Harrell FE Lee KL Califf RM Pryor DB Rosati RA. Regression modelling strategies for improved prognostic prediction. Stat Med (1984) 3(2):143–52. doi: 10.1002/sim.4780030207 6463451

