# Supplementary material for: Predicting head and neck cancer treatment outcomes with pre-treatment quantitative ultrasound texture features and optimising machine learning classifiers with texture-of-texture features
Source: Front Oncol. 2023 Oct 2;13:1258970. doi: 10.3389/fonc.2023.1258970 (PMC10578955; doi:10.3389/fonc.2023.1258970)
Supplement: Supplementary file 1 [file Image_1.pdf]

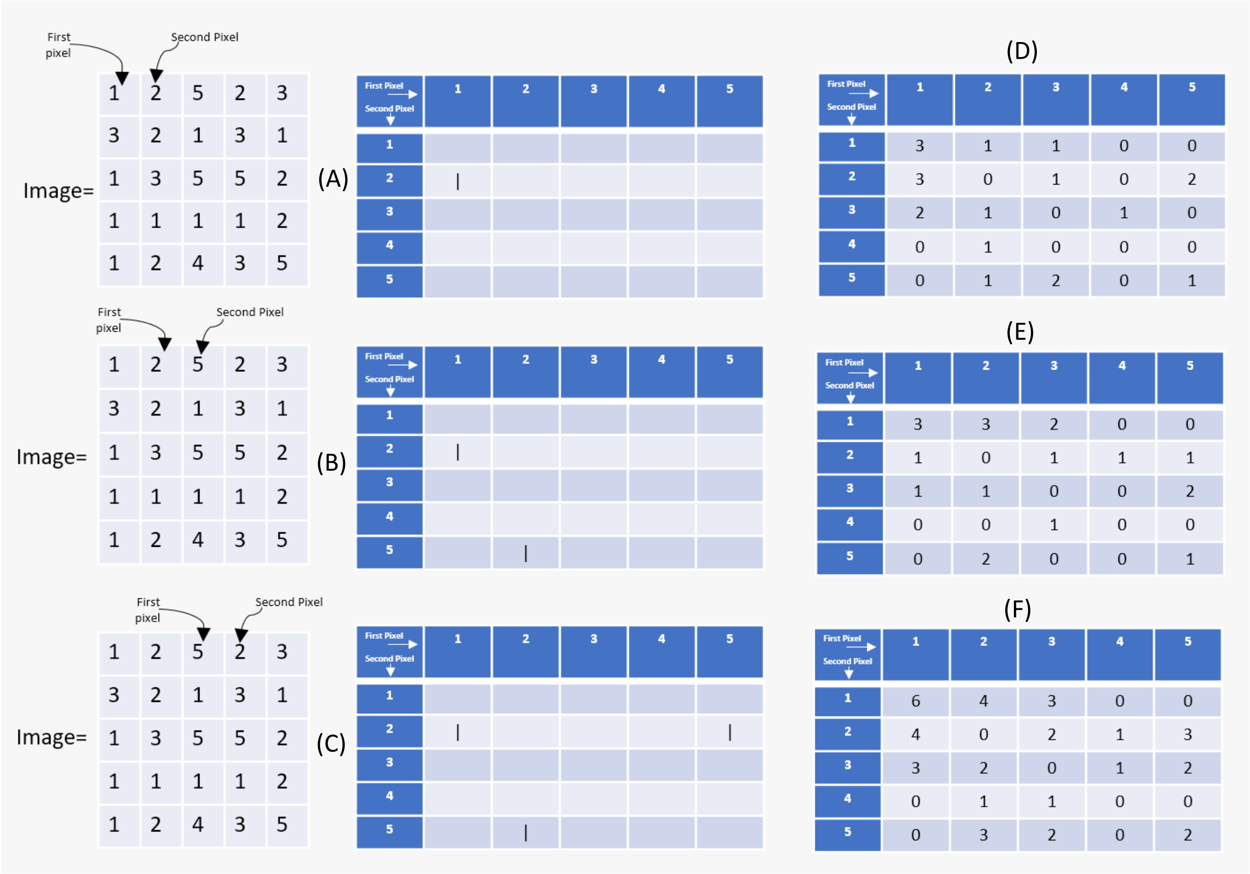

Supplementary Figure 1. Example of GLCM calculation. In (A) the first pixel has an intensity of 1 and the second pixel has an intensity of 2. The relationship between these neighbouring pixels is recorded in the GLCM on the right. In the second step (B) the first pixel has an intensity of 2 and the second pixel has an intensity of 5. The following step (C) shows a first pixel value of 5 and a second pixel value of 2. The GLCM is calculated one pixel at a time movie from left-to-right until all pixels have been considered as the 'first' pixel. The tally of the left-to-right GLCM can be seen in (D). (E) shows the right-to-left GLCM, and (F) shows the summation of (D) and (E). It should be noted that for more accuracy, a top-to-bottom GLCM and bottom-to-top GLCM should also be calculated and added to (F), but not shown here for brevity.
