# Supplementary material for: Predicting head and neck cancer treatment outcomes with pre-treatment quantitative ultrasound texture features and optimising machine learning classifiers with texture-of-texture features
Source: Front Oncol. 2023 Oct 2;13:1258970. doi: 10.3389/fonc.2023.1258970 (PMC10578955; doi:10.3389/fonc.2023.1258970)
Supplement: Supplementary file 2 [file Table_1.docx]

Supplementary Material

| **HN#**  *Supplementary Table 1 – Individual Patient characteristics of interest* | **Response** | **Age** | **Sex** | **Laterality** | **Biopsy description of disease** | **Location (Primary Mass)** | **HPV Status** | **Stage** | **Lymph Node** | **Chemotherapy Type** | **Radiation regimen** | **Smoking Habit** | **Alcohol Consumption** |
| --- | --- | --- | --- | --- | --- | --- | --- | --- | --- | --- | --- | --- | --- |
| HN 01 | **PR** | 52 | M | Right Neck | Loc. Adv. Squamous cell | Base of Tongue | P16+ | T3 N2c | Level III | Cisplatin | 70gy 33 fractions | lifetime non smoker | occasional drinker |
| HN 02 | **PR** | 54 | M | Left Neck | EBV positive non-keratinizing Nasopharyngeal Carcinoma | Nasopharynx | Unknown | not reported | Level II | Cisplatin | 70gy 33 fractions | lifetime non smoker | occasional drinker |
| HN 04 | **PR** | 54 | M | Right Neck | Squamous cell | Right tonsil | p16+ | cT2 N2b | Level II | Cisplatin | 70gy 33 fractions | 30-pack year | occasional drinker |
| HN 05 | **CR** | 59 | M | Left Neck | Squamous cell | Left Tonsil | p16+ | cT1 N2b | Level II | Cisplatin | 70gy 33 fractions | non smoker | non-drinker |
| HN 06 | **CR** | 82 | M | Left Neck | small-cell carcinoma | Left Parotid | Not reported | Not reported | Level II | Carboplatin+ Etoposide | 70gy 33 fractions | Not reported | Not reported |
| HN 07 | **PR** | 76 | M | Left Neck | metastatic Squamous cell | Bilateral Hypopharynx / Supraglottic Base of Tongue | p16+ | T2 N2b | Level IIa | Carboplatin | 70gy 33 fractions | 22-pack year (quit for 15 years) | non-drinker |
| HN 08 | **PR** | 62 | M | Right Neck | Poorly Differentiated Squamous cell | Supraglottic Larynx | Not reported | T2 N2c | Level II | Cisplatin | 70gy 33 fractions | non smoker | occasional drinker |
| HN 09 | **CR** | 74 | F | Right Neck | Squamous cell | Right Tonsil | p16+ | cT2 N2b | Level II / III | No chemo | 70gy 33 fractions | non smoker | non-drinker |
| HN 10 | **CR** | 68 | M | Left Neck | squamous cell | Left Glossotonsilar Sulcus | p16+ | not reported | Level II / III | Cisplatin | 70gy 33 fractions | 10-pack year | 4-5 drinks / week |
| HN 11 | **PR** | 59 | M | Left Neck | Squamous cell | Left Tonsil | p16+ | N2a | Level II | Cisplatin | 70gy 33 fractions | 45-pack year (quit in 2015) | 1-2 drinks/ day |
| HN 12 | **PR** | 57 | M | Left Neck | metastatic Squamous cell | unknown primary of the left neck | p16+ and p63+ | N2 | Level IIA | Cisplatin | 70gy 33 fractions | 10-pack year | occasional drinker |
| HN 14 | **CR** | 52 | M | Left Neck | Squamous cell | unknown primary of the left neck | Not reported | T4bN2c | Not reported | No Chemo | 70gy 33 fractions | 30 pack/yr | 6 per day |
| HN 15 | **PR** | 55 | M | Right Neck | Squamous cell | Presented with metastatic SCC (Locally advanced oropharyngeal Ca.) - involvement of Naso & Cribriform Plate | Not reported | T1N2 | Not reported | Cisplatin | 70gy 33 fractions | 10 pack year | 4-5 per week |
| HN 16 | **PR** | 68 | F | Left Neck | Squamous cell | Not reported | Tonsil | T3N2b | Not reported | Cisplatin | 69.96Gy 33 fractions | 5 pack year | 1 per day |
| HN 18 | **PR** | 61 | M | Right Neck | Squamous cell | Right Tonsil | p16+ | cT2 N2b | Level II and III | Cisplatin | 69.96Gy 33 fractions | 30-pack year | occasional drinker |
| HN 19 | **PR** | 60 | M | Right Neck | metastatic Squamous cell | unknown primary of the Right Neck | p16- | not reported | Level II / III | No chemo | 69.96Gy 33 fractions | 40-pack year | previous history of alcohol abuse but discontinued for 25 years |
| HN 20 | **CR** | 70 | M | Right Neck | Squamous cell | Right Base of Tongue | p16+ | T2 N2a | Level IIa / III | Cisplatin | 69.96Gy 33 fractions | 15-pack year | 6-12 beers / week |
| HN 21 | **CR** | 64 | M | Left Neck | Squamous cell | Larynx | not mentioned | T3 N1 | Level III | Cisplatin | 69.96Gy 33 fractions | 40-pack year | occasional drinker |
| HN 22 | **PR** | 57 | M | Left Neck | Squamous cell | Hypopharynx | not tested | T2 N3 | Level II / III | Cisplatin | 69.96Gy 33 fractions | 80-pack years | 10 drinks / day |
| HN 23 | **PR** | 66 | M | Right Neck | Squamous cell | Right Tonsil | p16+ | T2 N2b | Level II | Cisplatin | 69.96Gy 33 fractions | non smoker | occasional drinker |
| HN 24 | **PR** | 73 | M | Right Neck | Metastatic Squamous cell | Larynx | not tested | T3 N3 M1 | Level II | No chemo | 69.96Gy 33 fractions | 50-pack year | Heavy drinker |
| HN 25 | **PR** | 60 | M | Left Neck | Metastatic Squamous cell | Left Glossotonsillar Sulcus Base of Tongue | p16+ | T4 N2b | Level II | Cetuximab | 69.96Gy 33 fractions | 6-pack years | occasional drinker |
| HN 26 | **PR** | 40 | M | Right Neck | Squamous cell | Right Oropharynx | Not Reported | T4b N2c | Level II | Cisplatin | 69.96Gy 33 fractions | non-smoker | non-drinker |
| HN 27 | **PR** | 61 | M | Left Neck | Squamous cell | Base of Tongue | p16+ | not reported | Level II/ III | Cisplatin | 69.96Gy 33 fractions | 30-pack year | 10 drinks / week |
| HN 28 | **PR** | 59 | M | Left Neck | Squamous cell | Left Oropharynx | unclear | T4 N3 M0 | Level II | Cisplatin | 69.96Gy 33 fractions | non smoker | non-drinker |
| HN 29 | **PR** | 46 | F | Right Neck | Squamous cell | Right Tonsil | p16+ | T2/T3 N2 | Level II | Cisplatin | 69.96Gy 33 fractions | 8-pack year (quit 10 yrs ago) | Not reported |
| HN 30 | **CR** | 62 | M | Right Neck | squamous cell | Right Base of Tongue | p16+ | T2 N2 | Level Iib | Cisplatin | 69.96Gy 33 fractions | 20-pack year | 6 beers / night |
| HN 31 | **CR** | 54 | M | Right Neck | Squamous cell | Aryepiglottic fold | p16+ | T2 N2 M0 | Level II/ III | Cisplatin | 69.96Gy 33 fractions | 10 pack year | 2 drinks / day |
| HN 32 | **PR** | 58 | M | Right Neck | Squamous cell | Glossotonsillar Sulcus | not reported | not reported | Level II | Cisplatin | 69.96Gy 33 fractions | 10-pack year +marijuana (quit 10 yrs ago) | previously drank heavily (quit for 20) |
| HN 33 | **PR** | 52 | M | Left Neck | Squamous cell | Left Base of Tongue (Tonsillar Base) | p16+ | not mentioned | Level II/III | Cisplatin | 69.96Gy 33 fractions | 17-pack year | 12-20 beers / weekend for 20 years |
| HN 34 | **CR** | 75 | M | Right Neck | squamous cell | Posterior Pharyngeal | not mentioned | T2 N2a | Level II | No chemo | 69.96Gy 33 fractions | former smoker (quit for 50 years) | 3/ 4 beers / week |
| HN 35 | **CR** | 71 | M | Right Neck | Squamous cell | Right tonsillar and Base of tongue | p16+ | T2 N2b | Level IIa | Cisplatin | 69.96Gy 33 fractions | 2nd hand smoke exposure | 3-4 glasses of wine / day |
| HN 36 | **CR** | 49 | M | Left Neck | Squamous cell | Left Tonsil | p16+ | not mentioned | Level II | No chemo | 69.96Gy 33 fractions | Lifetime non-smoker | 4-5 glasses of wine / week |
| HN 37 | **CR** | 67 | M | Right Neck | Squamous cell | Ipsilateral Nasopharynx base of tongue | p16+ | T3 N2c | Level II a/b | Cisplatin | 69.96Gy 33 fractions | 2 packs a day (quit 1986) | Heavy alcohol use (quit 20 yrs ago) |
| HN 39 | **PR** | 59 | M | Right Neck | Squamous cell | Right Tonsil (Oropharynx) | p16+ | T2 N2b | Level II | high dose cisplatin (1 cycle) + 2 cycles carboplatin | 69.96Gy 33 fractions | Lifetime non-smoker | Lifetime non drinker |
| HN 40 | **CR** | 51 | M | Right Neck | squamous cell | BOT | HPV+ | T2N3B | unknown | Cisplatin | 69.96Gy 33 fractions | non smoker | Socially |
| HN 42 | **PR** | 64 | M | Left Neck | Squamous cell | Base of Tongue | p16+ | T2 N2a | Level IIa | Cisplatin | 69.96Gy 33 fractions | Ex-smoker quit in 2016 (50-pack-years) | 26 ounces of rum + beer |
| HN 43 | **PR** | 51 | M | Right Neck | Squamous cell | Base of Tongue | p16+ | not mentioned | Level II | Cisplatin | 69.96Gy 33 fractions | 40-pack-year (quit 2015) | 10 drinks |
| HN 44 | **PR** | 61 | M | Right Neck | moderately differentiated Squamous cell | Right Tonsil | p16+ | T1 N2c | Level IIa | Cisplatin | 69.96Gy 33 fractions | 35-pack-year | 5-drinks a day (for years) |
| HN 45 | **PR** | 59 | M | Left Neck | invasive poorly differentiated Squamous cell | Base of Tongue | p16- | T4 N3 | Level II and III | Cisplatin | 67.84Gy 32 fractions | Heavy smoker | Binge drinker |
| HN 46 | **PR** | 44 | F | Right Neck | Locally Advanced Squamous cell | Base of Tongue | Not reported | Not reported | Level IIA and III | Cisplatin | 69.96Gy 33 fractions | 10-pack-year | non-drinker |
| HN 47 | **PR** | 57 | M | Left Neck | Squamous cell | Left Glossotonsillar Sulcus | p16+ | T2 N1 | Level IIa | high dose cisplatin (1 cycle) and 1 cycle Carboplatin (due to tinnitus from cisplatin) | 69.96Gy 33 fractions | 20-pack-year (quit in 1997) | marijuana 50mg/day, 8-10 drinks |
| HN 48 | **PR** | 70 | M | Right Neck | poorly differentiated Squamous cell | Right Base of Tongue (Oropharynx) | p16+ | T2 N2 | Level IIA | Carboplatin AUC 6 ( due to tinnitus) | 69.96Gy 33 fractions | 16-pack-year (quit in 1980) | 2 alcoholic drinks |
| HN 49 | **PR** | 69 | M | Right Neck | poorly differentiated nonkeratnizing Squamous cell | Right Tonsil (Oropharynx) | p16+ | Not reported | Level II | cisplatin low dose (weekly) | 69.96Gy 33 fractions | 10-pack-year (quit in 1987) | < than 1 glass per week |
| HN 50 | **CR** | 63 | M | Right Neck | Squamous cell | Right Tonsil | p16+ | T2 N1 | Level II a - b | Carboplatin AUC 6 ( due to tinnitus) | 69.96Gy 33 fractions | Lifetime non-smoker | once a month |
| HN 51 | **PR** | 39 | M | Right Neck | metastatic nasopharyngeal carcinoma non keratinizing, undifferentiated (EBV positive) | Nasopharynx | Not reported | IVc | Level II a - b | Gemcitabine/Cisplatin (4 cycles) + Cisplatin low dose (8 cycles) | 65.72Gy 31 fractions | 10-pack-year | non-drinker |
| HN 52 | **PR** | 63 | M | Right Neck | Invasive Squamous cell | Right Piriform Fossa and of the Right Aryepiglottic Fold | Not reported | Not reported | Level II | Cisplatin | 69.96Gy 33 fractions | 45-pack-year (quit June 2017) | several beers / day |
| HN 53 | **PR** | 65 | M | Left Neck | Squamous cell | Base of Tongue | p16+ | T3 N2 | Level II - III | Cisplatin | 69.96Gy 33 fractions | cigars for 30 years | 24 beers / week |
| HN 54 | **CR** | 50 | M | Left Neck | Squamous cell | Base of Tongue | p16+ | T1 N2b | Level IIb - III | Cisplatin | 69.96Gy 33 fractions | Lifetime Non-smoker | Not specified |
| HN 55 | **PR** | 79 | M | Left Neck | Squamous cell | Unknown primary of the left neck mass | Not reported | Not reported | Level II | No chemo | 69.90Gy 30 fractions | Lifetime Non-smoker | occasional social drinker |
| HN 56 | **PR** | 39 | M | Left Neck | Poorly differentiated metastatic Squamous cell carcinoma | Left Tonsil | p16+ | Not reported | level II | Cisplatin | 69.96Gy 33 fractions | ex-smoker quit in 2014 (7.5-pack-years) | occasional social drinker |
| HN 57 | **PR** | 56 | M | Right Neck | Locally advanced metastatic nasopharyngeal carcinoma | Nasopharynx | EBV positive | T2N1 | Level II - level V | Carboplatin | 69.96Gy 33 fractions | Ex-smoker 8 cig / day (quit 15-20 years ago) | 1 glass wine/day |
| HN 58 | **PR** | 58 | M | Right Neck | Invasive squamous cell carcinoma of unknown primary | unknown primary (suspected BOT) | Not reported | Not reported | level IIA and B | Cisplatin | 69.96Gy 33 fractions | Occasional cigars over 15 years | Six to eight drinks per week |
| HN 59 | **CR** | 58 | M | Right Neck | Squamous Cell Carcinoma | Base of Tongue | p16+ | T4 N2 | level II | Cisplatin | 69.96Gy 33 fractions | Never smoked | Not reported |
| HN 60 | **CR** | 80 | M | Right Neck | Poorly Differentiated Squamous cell carcinoma | Right Tonsil | p16+ | T2 N2 | Level II | No Chemo | 69.96Gy 33 fractions | Smoked for 3 years (0.5 to 1 pack per day) | occasional drinker |
| HN 63 | **PR** | 44 | M | Left Neck | Squamous cell carcinoma | Mandibular mucosa, left mandible body, buccal mucosa, masseter, medial pterygoid muscle, floor of mouth, mylohyoid, root of tongue including genioglossus, glossotonsillar sulcus, soft palate, lateral oropharyngeal wall | Not reported | T4 N2c | Level I - IV | Cisplatin | 69.96Gy 33 fractions | Lifetime non-smoker | Minimal consumption |
| HN 64 | **PR** | 76 | M | Left Neck | Squamous cell carcinoma with bilateral neck involvement | Base of Tongue | Not reported | T4 N2 | Not reported | No chemo | 69.96Gy 33 fractions | Lifetime non-smoker | 2-3 drinks/week |
| HN 65 | **CR** | 65 | M | Left Neck | Squamous cell carcinoma | Base of Tongue | Not reported | T4a N2 | Level IIa/III | Cisplatin | 69.96Gy 33 fractions | Lifetime non-smoker | 3-4 drinks/day |
| HN 66 | **CR** | 47 | M | Left Neck | Squamous Cell Carcinoma | Oropharynx | p16+ | T4 | Not reported | Cisplatin | 69.96Gy 33 fractions | Lifetime non-smoker | Not reported |
| HN 67 | **PR** | 66 | M | Left Neck | Squamous Cell Carcinoma | Oropharynx | p16+ | T4 | Level IB | Cisplatin | 69.96Gy 33 fractions | Smoker; quit in 2009 | 50 drinks/week |
| HN 68 | **PR** | 61 | F | Left Neck | Squamous Cell Carcinoma | Nasopharynx | Not reported | T4 | Level IIA | No Chemo | 69.96Gy 33 fractions | Smoker - 40packs/year | Not reported |
| HN 69 | **CR** | 56 | M | Right Neck | Squamous Cell Carcinoma | Hypopharynx | Not reported | T4 | Level Iia+b | Cisplatin | 69.96Gy 33 fractions | Smoker - 30packs/year | Socially |
| HN 70 | **CR** | 67 | M | Right Neck | Squamous Cell Carcinoma | Unknown primary | p16+ | T0 | N1 | Cisplatin | 69.96Gy 33 fractions | Quit smoking (1pack/year for 20 yrs) | Socially |
| HN 71 | **CR** | 70 | M | Right Neck | Squamous Cell Carcinoma | Right BOT | p16+ | T4 | N1 | Cisplatin | 69.96Gy 33 fractions | Quit smoking (50pack/year) | Socially |
| HN 73 | **CR** | 71 | M | Right Neck | Squamous Cell Carcinoma | Supraglottic laryngeal | Not reported | T3 | N2c | Cisplatin | 70Gy 35 fractions | nonsmoker | non-alcoholic |
| HN 74 | **PR** | 78 | M | Right Neck | Squamous Cell Carcinoma | Right Tonsillar | p16+ | T2 | N1 | Cisplatin | 69.96Gy 33 fractions | non-smoker | non-alcoholic |
| HN 75 | **CR** | 68 | M | Right Neck | Squamous Cell Carcinoma | Right soft palate | p16+ | Not reported | Not reported | Cisplatin | 69.96Gy 33 fractions | Smoker (45 pack/year) | non-alcoholic |
| HN 76 | **PR** | 65 | M | Right Neck | Squamous Cell Carcinoma | Oropharynx | p16+ | T2 | N1 | Cisplatin | 70Gy 35 fractions | Smoker (2 pack/year) | 18 glasses per week |
| HN 77 | **PR** | 53 | M | Right Neck | Squamous Cell Carcinoma | Oropharynx | Not Reported | T0/N3 | L11 | Cisplatin | 70Gy 33 fractions | 1/4 pack/day since age of 20 - quit two weeks ago | non-alcoholic |
| HN 78 | **PR** | 36 | M | Right Neck | Nasopharyngeal Carcinoma | Not reported | EBV positive | Not reported | Level IIA & IIB | Cisplatin (high dose) | 70Gy 33 fractions | Ex-smoker (2 packs/year-quit 1 year ago) | non-alcoholic |
| HN 79 | **PR** | 55 | M | Right neck | Squamous cell carcinoma | Base of tongue | p16+ | T4 | N2 | Cisplatin | 70Gy 33 fractions | Nonsmoker | non-alcoholic |
| HN 80 | **PR** | 67 | M | Left Neck | Squamous cell carcinoma | Unknown primary | Not reported | Not reported | Level IIB | Carboplatin | 70Gy 33 fractions | Nonsmoker | 4 drinks per week |
